# Temporal Clustering of *Mycoplasma pneumoniae*–Associated Encephalitis and Stroke, South Korea, 2024

**DOI:** 10.3201/eid3202.251296

**Published:** 2026-02

**Authors:** Seung Ha Song, Dayun Kang, Ye Kyung Kim, Jaeso Cho, Hye Jin Kim, Woojoong Kim, Ki Wook Yun

**Affiliations:** SMG-SNU Boramae Medical Center, Seoul, South Korea (S.H. Song, H.J. Kim); Seoul National University College of Medicine, Seoul (S.H. Song, K.W. Yun); Seoul National University Children’s Hospital, Seoul (D. Kang, W. Kim, K.W. Yun); Seoul National University Bundang Hospital, Seoul National University College of Medicine, Seongnam, South Korea (Y.K. Kim, J. Cho)

**Keywords:** bacteria, Mycoplasma pneumoniae, respiratory infections, meningitis/encephalitis, stroke, children, South Korea

## Abstract

Seventeen pediatric encephalitis (n = 12) or stroke (n = 5) cases clustered temporally during the 2023–2024 *Mycoplasma pneumoniae* epidemic in South Korea; similar patterns had not been noted in previous seasons. Those findings might reflect postpandemic changes in clinical manifestation and underscore the need for neurologic surveillance during *M. pneumoniae* epidemics.

*Mycoplasma pneumoniae* is a major cause of community-acquired pneumonia in children and can also lead to extrapulmonary complications affecting multiple organ systems, including the central nervous system (CNS) (*1*,*2*). Neurologic complications, including encephalitis and stroke, are clinically consequential because of their severity and sequelae.

During the COVID-19 pandemic, nonpharmaceutical interventions markedly reduced the circulation of common respiratory pathogens, including *M. pneumoniae* (*3*). However, since 2023, a resurgence of *M. pneumoniae* has been reported worldwide (*4*). Amid this resurgence, we observed increased numbers of pediatric encephalitis and ischemic stroke cases associated with *M. pneumoniae* in South Korea during the 2023–2024 season (*5*). To better characterize that phenomenon, we analyzed the clinical features and outcomes of affected children and evaluated institutional and national surveillance data to place those findings in broader temporal and epidemiologic contexts.

## The Study

We conducted a retrospective study of children hospitalized with encephalitis or ischemic stroke at 2 tertiary hospitals (center A, Seoul; center B, Seongnam) and 1 secondary care hospital (center C, Seoul) during the October 2023–December 2024 *M. pneumoniae* epidemic in South Korea, as defined by the Korea Disease Control and Prevention Agency surveillance program (Appendix Figure 1). *M. pneumoniae* infection was confirmed by using PCR of respiratory samples or by using serology (particle agglutination titer >1:160 or IgM index >1.4 by using enzyme-linked immunosorbent assay) (*6*). We defined encephalitis as an altered mental status lasting >24 hours with supportive tests and stroke as focal deficits with radiographic infarctions.

**Figure 1:**
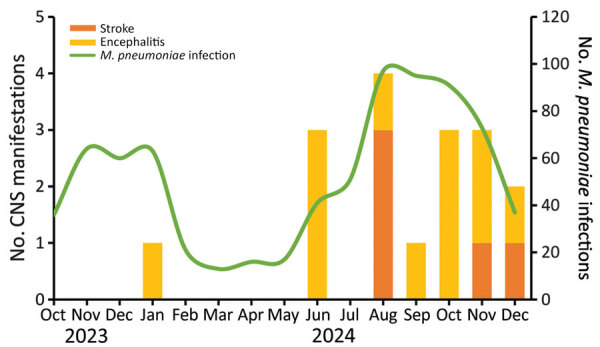
Monthly pediatric stroke and encephalitis cases (stacked bars) and *Mycoplasma pneumoniae* infections (green line) on the basis of aggregated data obtained from 3 hospitals in South Korea, October 2023–December 2024. Scales for the y-axes differ substantially to underscore patterns but do not permit direct comparisons.

We identified 17 cases of *M. pneumoniae*–associated encephalitis (n = 12) or ischemic stroke (n = 5). Of those cases, 13 occurred at center A, 4 at center B, and none at center C. Most cases were clustered during June–December 2024, which coincided with a marked increase in *M. pneumoniae* infections (Figure 1).

The median patient was 8.8 years of age; 52.9% of the patients were male and 47.1% female. Although 4 patients had no preceding respiratory symptoms, pneumonia was documented in 11 patients. The median interval from respiratory to neurologic symptom onset was 3 days (range 2–8 days). *M. pneumoniae* infection was confirmed in all 17 patients; 10 cases were confirmed by using both PCR (respiratory specimens) and serology, 6 cases by using serology, and 1 by using PCR (respiratory specimen). Cerebrospinal fluid PCR was performed on samples from 2 patients (1 positive case and 1 indeterminate case), and cerebrospinal fluid serology for *M. pneumoniae* was not performed. No other infectious or autoimmune etiologies were detected in the 17 patients. All 5 stroke patients had large-vessel infarctions in the middle cerebral artery or anterior cerebral artery territories; moreover, the clinical manifestations of 4 patients included multiterritorial involvement. Brain magnetic resonance imaging abnormalities (typically involving diffusion restriction or T2-weighted fluid-attenuated inversion recovery hyperintensities in the hippocampus, basal ganglia, or thalamus) were identified in 42% of the encephalitis patients.

Seven patients required intensive care unit admission. Twelve patients received intravenous antimicrobial drugs and 9 received corticosteroids; in addition, intravenous immunoglobulin was administered to 4 encephalitis patients. The outcomes were favorable; at the last follow-up, 83.3% of encephalitis and 60.0% of stroke patients demonstrated a modified Rankin scale score <2 (0–2 = no to mild disability), and no deaths were reported (Appendix Table 1).

During the same period (October 2023–December 2024), we identified 193 *M. pneumoniae*–associated hospitalizations without CNS involvement at the 3 hospitals for comparison. The median age and proportion of male patients were similar between the groups; however, the CNS complication group demonstrated substantially longer hospital stays (29 vs. 6 days; p<0.001) and a higher intensive care unit admission rate (41% vs. 3%; p<0.001) (Appendix Table 2).

**Figure 2:**
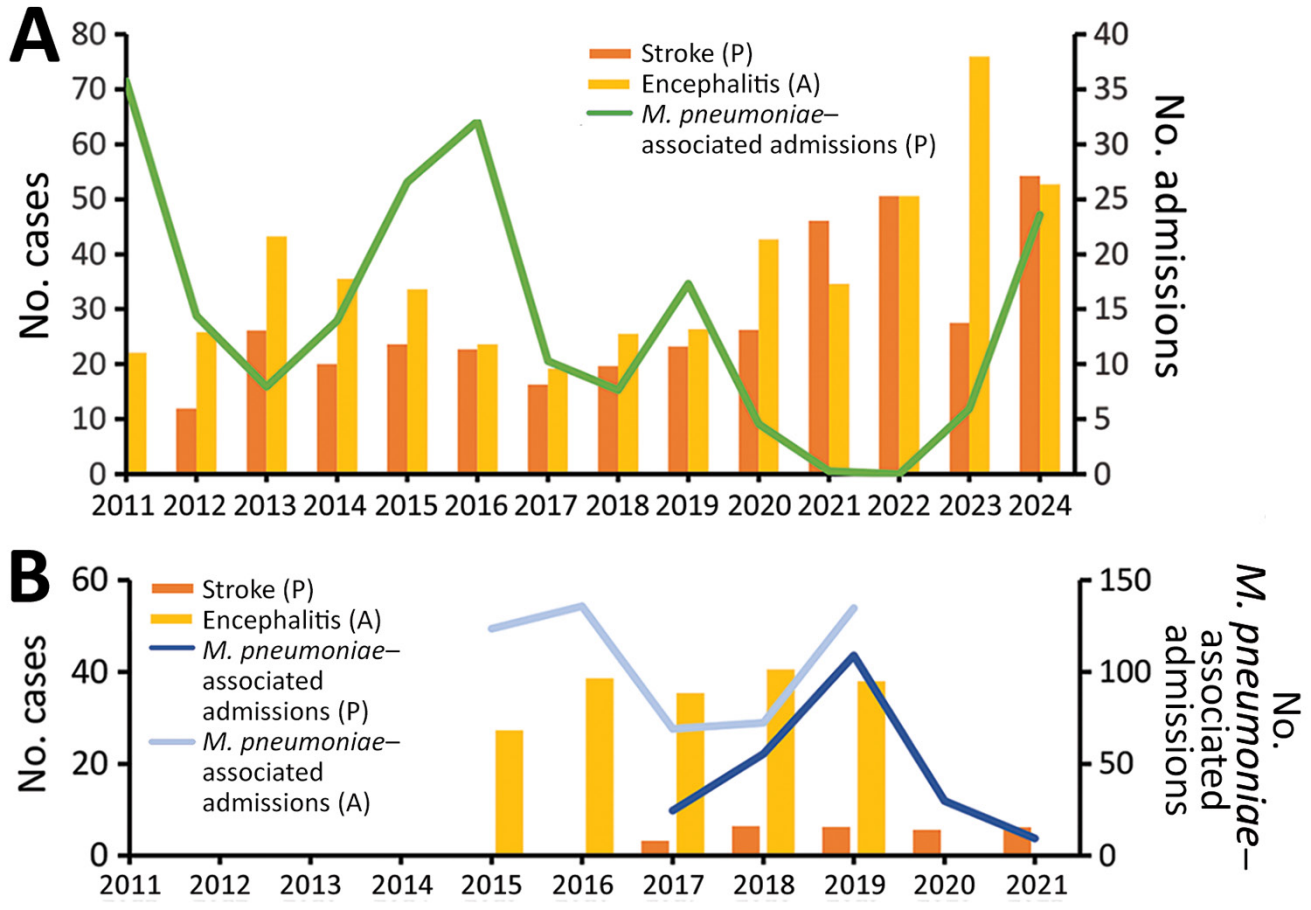
Annual trends in pediatric *Mycoplasma pneumoniae*–associated admissions and central nervous system complications in South Korea. A) Data obtained from 3 hospitals during 2011–2024. Bars represent ischemic stroke and encephalitis cases per 10,000 pediatric hospitalizations. Data line indicates annual *M. pneumoniae*–associated pediatric admissions per 1,000 hospitalizations. B) National data from 2015–2021. Bars show annual pediatric stroke and all-age encephalitis cases. Data lines represent *M. pneumoniae*–associated hospitalizations in pediatric and all-age populations reported to the Korea Disease Control and Prevention Agency sentinel surveillance system. All values in panel B are presented on a 1/100 scale. A, all age; P, pediatric. Scales for the y-axes differ substantially to underscore patterns but do not permit direct comparisons.

**Figure 3:**
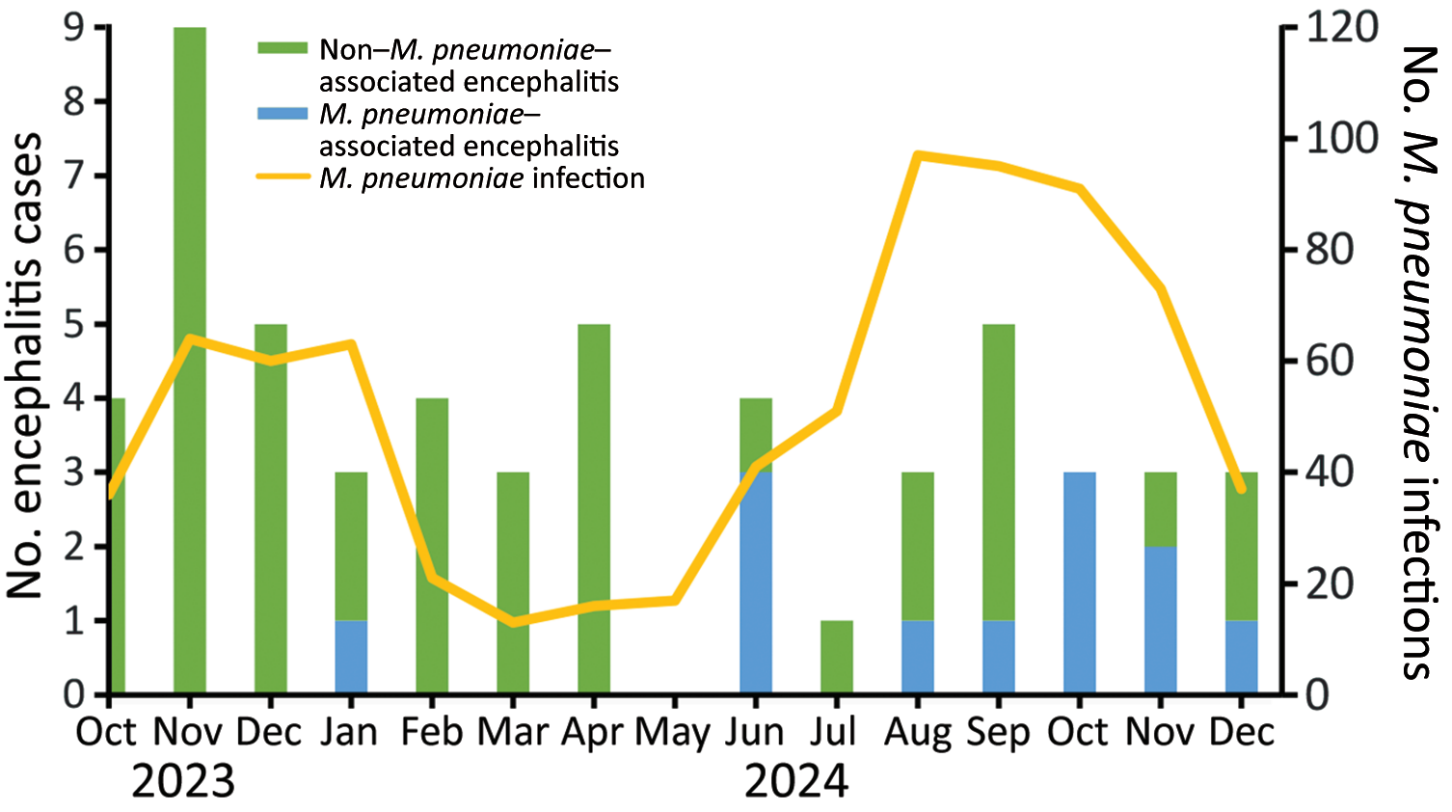
Monthly encephalitis cases by *Mycoplasma pneumoniae* association during the 2023–2024 epidemic, with concurrent *M. pneumoniae* infections (October 2023–December 2024). The bars show monthly encephalitis hospitalizations aggregated across 3 hospitals, which were divided into *M. pneumoniae*–associated and non–*M. pneumoniae*–associated cases. Data line denotes the number of *M. pneumoniae* infections identified at the 3 hospitals.

We analyzed data from 2011–2024 to evaluate long-term trends in pediatric stroke, encephalitis, and *M. pneumoniae*–associated hospitalizations, which were identified by using administrative diagnostic codes from standardized clinical data warehouse records; in addition, we normalized annual case counts by total pediatric admissions for comparison. At the institutional level, the stroke and encephalitis rates did not consistently align with *M. pneumoniae* epidemic activity; however, both rates increased after 2020, possibly reflecting broader effects of the COVID-19 pandemic and its aftermath (Figure 2, panel A). In contrast, a distinct temporal overlap emerged in 2024; during June–December, *M. pneumoniae* infections accounted for 20%–100% of monthly encephalitis admissions across sites (Figure 3), suggesting a temporal association between *M. pneumoniae* circulation and neurologic complications during the epidemic.

We assessed nationwide trends in *M. pneumoniae*-associated admissions, encephalitis, and pediatric stroke from public datasets. We obtained *M. pneumoniae*–associated hospitalization data from the Korea Disease Control and Prevention Agency sentinel surveillance program (*5*), pediatric stroke data from the Korean Statistical Information Service (*7*), and nationwide encephalitis data from health insurance claims, excluding cases with confirmed pathogens (*8*). The incidence of encephalitis (all ages) remained stable from 2015–2019, with no apparent temporal association being detected with *M. pneumoniae* activity. In addition, the pediatric stroke data obtained from 2017–2021 did not demonstrate a temporal relationship with monthly *M. pneumoniae* activity (Figure 2, panel B).

The absence of temporal associations during previous epidemics contrasts with the clear increase in *M. pneumoniae*–associated CNS cases in 2024. That pattern might be partially explained by the immunity gap hypothesis (*9*), whereby prolonged suppression of respiratory pathogens during COVID-19 nonpharmaceutical interventions increased pediatric susceptibility, which is consistent with shifts in age distribution and disease severity reported in other studies (*10*,*11*). *M. pneumoniae* neurologic complications might result from direct CNS invasion, immune-mediated damage, and cerebrovascular occlusion *(**12*). In this context, an increase in primary *M. pneumoniae* infections among seronegative children during 2023–2024 plausibly increased the risk for immune-mediated CNS complications; however, that relationship warrants further investigation. Moreover, strain features might contribute to increased severity; in 2023–2024, several studies reported P1 type 1 predominance and widespread A2063G macrolide resistance, accompanied by a high proportion of severe pulmonary manifestations (*13*,*14*). Strains exhibiting greater neurotropism might have been circulating during this period, although further validation is needed. Increased clinical awareness and the large scale of the epidemic might have increased the absolute number of rare complications.

The first limitation of this study is that the use of serologic testing to confirm *M. pneumoniae* infection reveals known constraints, including potential false positivity, delayed antibody responses, and interassay variability. Second, nationwide datasets lacked monthly resolution and pediatric detail and originated from different sources (the Korean Statistical Information Service and insurance claims data), limiting comparability. Finally, the absence of pathogen characterization precluded the evaluation of strain-specific factors potentially associated with neurologic complications. 

## Conclusions

Despite those limitations, our study provides clinical data highlighting increased pediatric CNS manifestations of *M. pneumoniae* infection observed during the 2023–2024 epidemic. In contrast to prior years, we observed a distinct temporal clustering of *M. pneumoniae*–associated CNS manifestations in South Korea in 2024. It is unclear whether the observed pattern reflects diagnostic capacity, heightened clinical vigilance, or a true shift in disease dynamics potentially driven by host, pathogen, or environmental factors. Further large-scale and longitudinal studies are warranted to determine whether this clustering represents an isolated event or signals an emerging trend in pediatric infectious diseases. Our findings underscore the importance of sustained epidemiologic surveillance and molecular characterization of circulating strains to better elucidate the mechanisms underlying extrapulmonary complications of *M. pneumoniae* infection. 

Appendix Additional information about temporal clustering of *Mycoplasma pneumoniae*–associated encephalitis and stroke, South Korea, 2024.
