# Supplementary material for: Temporal Clustering of Mycoplasma pneumoniae–Associated Encephalitis and Stroke, South Korea, 2024
Source: Emerg Infect Dis. 2026 Feb;32(2):270–3. doi: 10.3201/eid3202.251296 (PMC12928221; doi:10.3201/eid3202.251296)
Supplement: Appendix — Additional information about temporal clustering of Mycoplasma pneumoniae–associated encephalitis and stroke, South Korea, 2024. [file 25-1296-Techapp-s1.pdf]

Article DOI: https://doi.org/10.3201/eid3202.251296

EID cannot ensure accessibility for supplementary materials supplied by authors. Readers who have difficulty accessing supplementary content should contact the authors for assistance.

Temporal Clustering of *Mycoplasma pneumoniae*–Associated Encephalitis and Stroke, South Korea, 2024

Appendix

Appendix Table 1. Clinical characteristics and outcomes of children with *Mycoplasma pneumoniae*–associated CNS complications*

No.	Group	Age (y)	Sex (M/F)	Underlying disease	MP pneumonia	Throat PCR	Serology	CSF WBC (Poly/Lymph/ Others)/Protein†	Initial neurologic symptoms	MRI findings (Involved area or vessels)	ICU admission	mRS	Follow-up Period (months)
1	Stroke	6.9	F	MMD	+	+	1:2560	ND	Lt. side weakness	Both frontal PVWM and Rt. basal ganglia (both distal ICAs)	No	0	4
2	Stroke	7.9	F	None	+	+	1:2560	ND	Headache, Lt. hemiplegia	Rt. Frontal lobe (Rt. MCA)	Yes	3	6
3	Stroke	2.7	M	None	+	+	1:1280	ND	Lt. hemiplegia, Rt. eyeball deviation	Rt borderzone area (Rt. MCA)	No	1	5
4	Stroke	11.3	F	None	-	-	1:160	11(P36L18O46)/28	Headache, Lt. side weakness	Rt. ACA/MCA territories, occlusion of the right distal ICA and diffuse narrowing of the right proximal ICA	Yes	5	8
5	Stroke	14.4	M	Dystonia	+	+	ND	ND	Lt. hemiplegia	CLOCC, Right BG infarction	No	0	1
6	Encephalitis	10.3	F	None	-	ND	1:320	0/20	Seizure, altered mentality	Diffuse brain atrophy, wide spread diffusion restrictions	Yes	3	2
7	Encephalitis	5.1	F	Ganglio-neuroblastoma	+	+	1:2560	10(L90/O10)/142	Dysarthria, altered mentality seizure	Diffusion restriction in bilateral hippocampus	Yes	0	5
8	Encephalitis	12.7	M	None	+	+	1:20480	6(P33/O67)/41	Altered mentality	Normal	Yes	1	4
9	Encephalitis	9.0	M	None	+	+	1:2560	374(P13L39O48)/91	Headache, dizziness altered mentality, diplopia	Normal	No	0	0.25
10	Encephalitis	13.8	F	None	+	-	1:640	81(L89O11)/70	Altered mentality, urinary incontinence	Subtle T2 high signal intensity in both BG, thalamus	No	0	5
11	Encephalitis	8.8	F	None	-	+	1:2560	414(P1L64O35)/77	Dizziness, altered mentality	T2 high signal intensity in Lt. thalamus, Rt. Parietal PVWM, bilateral cerebellar WM	No	0	3
12	Encephalitis	11.3	M	None	-	ND	1:320	197(P3L78O19)/60	Headache, altered mentality	Normal	No	1	0.5
13	Encephalitis	5.3	F	None	-	-	1:320	0/17	Ataxia	Diffusion restriction in bilateral hippocampus	No	0	3
14	Encephalitis	8.3	M	None	+	+	3.21	17(P2O98)/71	Seizure, altered mentality	Normal	Yes	0	7
15	Encephalitis	8.2	M	None	-	+	2.56	95(P5/O95)/90	Altered mentality	Normal	Yes	2	4
16	Encephalitis	7.8	M	None	+	+	4.01	111(P23/O77)/64	Altered mentality	Normal	No	0	0.5
17	Encephalitis	8.8	M	None	+	ND	3.54	59(P1/O99)/54	Altered mentality	Normal	No	0	0.5

*CNS, central nervous system; PCR, polymerase chain reaction; CSF, cerebrospinal fluid; MRI, magnetic resonance imaging; ICU, intensive care unit; mRS, modified Rankin Scale; y, years; M, male; F, female; MP, *Mycoplasma pneumoniae*; MMD, moyamoya disease; WBC, white blood cell; P, polymorphonuclear cells; L, lymphocytes; O, other cells (e.g., monocytes, eosinophils); ACA, anterior cerebral artery; MCA, middle cerebral artery; ICA, internal carotid artery; BG, basal ganglia; CLOCC, cytotoxic lesion of the corpus callosum; PVWM, periventricular white matter; WM, white matter; +, positive; –, negative; ND, not done.

†CSF findings are presented as total white blood cell (WBC) count (cells/µL), followed by the percentage distribution of polymorphonuclear cells (predominantly neutrophils), lymphocytes, and other cells (mainly monocytes), and protein concentration (mg/dL). Percentage sums to 100%. Red blood cells were not included in WBC counts.

Appendix Table 2. Comparison of *M. pneumoniae*-associated hospitalizations with and without CNS involvement, 2024*

Category	CNS (n=17)	Non-CNS (n=193)	*P* value†
Age (y), median (IQR)	8.8 (7.8-11.3)	8.2 (5.6-11.1)	0.28
Gender (male), n (%)	9 (52.9)	99 (51.3)	0.73
Length of stay, days, median (IQR)	29 (14-52)	6 (4-9)	< 0.001
ICU admission, n (%)	7 (41.2)	6 (3.1)	< 0.001

* CNS, central nervous system; y, years; IQR, interquartile range; ICU, intensive care unit
†*P* values calculated using Wilcoxon rank-sum test for continuous variables and χ^2 or Fisher's exact test for categorical variables.

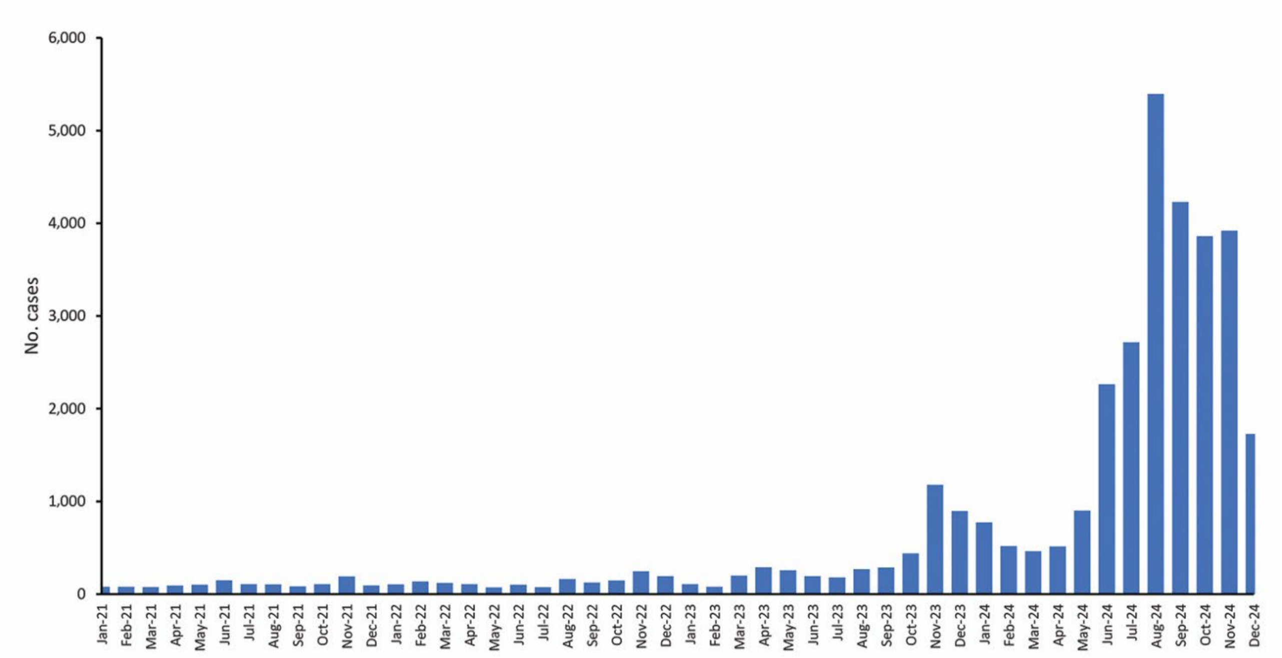

Appendix Figure. Monthly *Mycoplasma pneumoniae*–associated hospitalizations in South Korea, 2021–2024. Data obtained from the Korea Disease Control and Prevention Agency sentinel surveillance system.
